# Differentially Expressed Genes Analysis in the Human Small Airway Epithelium of Healthy Smokers Shows Potential Risks of Disease Caused by Oxidative Stress and Inflammation and the Potentiality of Astaxanthin as an Anti-Inflammatory Agent

**DOI:** 10.1155/2023/4251299

**Published:** 2023-03-03

**Authors:** Irandi Putra Pratomo, Aryo Tedjo, Dimas R. Noor, Rosmalena

**Affiliations:** ^1^Department of Pulmonology and Respiratory Medicine, Faculty of Medicine, Universitas Indonesia, Jakarta, Indonesia; ^2^Pulmonology and Respiratory Medicine Staff Group, Universitas Indonesia Hospital, Universitas Indonesia, Depok, Indonesia; ^3^Bioinformatics Core Facilities, Indonesian Medical Education and Research Institute, Faculty of Medicine, Universitas Indonesia, Jakarta, Indonesia; ^4^Drug Development Research Cluster, Indonesian Medical Education and Research Institute, Faculty of Medicine, Universitas Indonesia, Jakarta, Indonesia; ^5^Department of Medical Chemistry, Faculty of Medicine, Universitas Indonesia, Jakarta, Indonesia; ^6^Master's Programme in Biomedical Sciences, Faculty of Medicine, Universitas Indonesia, DKI Jakarta, Indonesia; ^7^Human Cancer Research Center, Indonesian Medical Education and Research Institute, Faculty of Medicine, Universitas Indonesia, Jakarta, Indonesia

## Abstract

Cigarette smoke (CS) was known for its effect of increasing oxidative stress that could trigger tissue injury and endothelial dysfunction mediated by free radicals and reactive oxygen species (ROS). ROS itself is a key signaling molecule that plays a role in the development of inflammatory disorders. Nuclear factor erythroid2 related factor2 (Nrf2) is the main regulator of antioxidant cellular response to cell and tissue-destroying components caused by CS. Nrf2 protein that is significantly activated in the smokers' small airway epithelium is followed by a series of gene expression changes in the same cells. This study aims to observe differentially expressed genes (DEGs) in the human small airway epithelium of smokers compared to genes whose expression changes due to astaxanthin (AST) treatment, an antioxidant compound that can modulate Nrf2. Gene expression data that was stored in the GEO browser (GSE 11952) was analyzed using GEO2R to search for DEG among smokers and nonsmokers subject. DEG was further compared to those genes whose expression changes due to astaxanthin treatment (AST) that were obtained from the Comparative Toxicogenomics Database (CTD; https://ctdbase.org/). DEG (*p* < 0.05) analysis result shows that there are 23 genes whose expression regulation is reversed compared to gene expression due to AST treatment. The gene function annotations of the 23 DEGs showed the involvement of some of these genes in chemical and oxidative stress, reactive oxygen species (ROS), and apoptotic signaling pathways. All of the genes were involved/associated with chronic bronchitis, adenocarcinoma of the lung, non-small-cell lung carcinoma, carcinoma, small cell lung carcinoma, type 2 diabetes mellitus, emphysema, ischemic stroke, lung diseases, and inflammation. Thus, AST treatment for smokers could potentially decrease the development of ROS and oxidative stress that leads to inflammation and health risks associated with smoking.

## 1. Introduction

Oxidative stress occurs due to an imbalance between the increased production of free radicals and decreased antioxidant capacity [1]. Under physiological conditions, oxidative stress will trigger an increase in the expression of endogenous antioxidant genes and cytoprotective proteins to prevent or limit tissue damage. This process is mediated by nuclear factor erythroid2 related factor2 (Nrf2) activity which then activates transcription way for antioxidant gene and enzyme detoxification [1, 2]. Thus, impaired activation of Nrf2 will cause a decrease in antioxidant capacity.

Cigarette smoke (CS) component that is dissolved in water is known to directly increase oxidative stress that could trigger tissue injury. Smoking tobacco has also been associated with vascular endothelium dysfunction through causative methods depending on the dose. This is mainly related to tobacco content of reactive oxygen species (ROS), nicotine, and inflammation driven by oxidative stress [3]. In particular, chronic CS exposure to respiratory tract tissue causes an increase in radical concentration, volatile compound (particularly oxygen species and reactive nitrogen), and CS condensate deposition, which will trigger a pleiotropic adaptive response, aimed at restoring tissue homeostasis [4]. Chronic exposure to CS generally encounters a cellular defense system characterized by activation of Nrf2. Nrf2 as the main regulator of antioxidant cellular response is proven to regulate the first line of defense against CS-induced cell and tissue-damaging components. This is indicated by the higher expression of Nrf2 in PBMC in moderate smokers compared to nonsmokers (*p* < 0.01). An increase in Nrf2 was not found in heavy smokers who possess a high level of nuclear transcription factor (NF-kB) and C-reactive protein (CRP) (*p* > 0.01) [5]. This indicates disruption of the Nrf2 role in heavy smokers with an inflammatory problem. Nrf2 genetic effect also affects smokers' health status. This is indicated by the significant interaction between genotype rs6726395 with accompanied by the decrease of forced expiratory volume in one second (FEV1) (*p*=0.011) [6]. The Haplotype rs2001350T/rs6726395A/rs1962142A/rs2364722A/rs6721961T is also associated with a lower annual decline in FEV1 (*p*=0.004) [6].

Astaxanthin (AST) is a food xanthophyll that is often found in sea organisms, and because of its unique molecular feature, it possesses good antioxidant activity. More evidence has suggested AST's protective role to counter several diseases where oxidative stress and inflammation occur continuously. AST is known to modulate Nrf2 binding to antioxidant response elements (AREs) in the promoter region of most cytoprotective or detoxifying enzymes [7]. Recent studies have also shown that AST modulates the NF-B signaling network by increasing inflammation and oxidative stress in various experimental models [8, 9]. Several studies have shown that the anti-aging effect, as well as attenuation of oxidative stress and inflammation of AST, is carried out through Nrf2 activation and NF-kB inhibition [10–12].

*Differentially Expressed Gene* (DEG) is important to understand the biological difference between a healthy and ill condition. Identification of genes involved in disease is an important tool for revealing the molecular mechanisms of disease development. In pharmaceutical and clinical studies, DEG also plays an important role to choose biomarker candidates, therapeutic targets, and genetic signatures for diagnosis [13]. In this study, an analysis of changes in gene expression patterns due to smoking was carried out in the Gene Expression Omnibus (GEO) database [14, 15] which was compared with changes in profile expression genes due to AST treatment obtained from *Comparative Toxicogenomics Database* (CTD; https://ctdbase.org/) [16]. By comparing these two research data, it is intriguing to know what genes have the potential expression to be affected by AST, so it is hoped that it could further explain the potential of AST as a candidate for antioxidant supplements in terms of its mechanism of action in reducing the health effects that can appear on smokers.

## 2. Methods

This study gathers data from *Gene Expression Omnibus* (GEO) database, a study conducted by Hübner et al. [17]. The inclusion criteria of a healthy nonsmoker and smoker referred to those study. Healthy nonsmoker was people with normal physical examination, lung function, and chest X-ray, with smoking-related blood and urine within the nonsmoker range. The criteria for a healthy smoker were current smoking history, followed by normal physical examination, lung function, chest X-ray, and smoking-related blood and urine parameters consistent with current smokers. In the study, the age of the subjects was not distinguished [17]. Human small airway epithelium samples was obtained using fiber-optic bronchoscopy of 38 healthy nonsmokers and 45 healthy smokers, and Nrf2-associated gene expression was assessed using the Affymetrix HG-U133 Plus 2.0 microarray. Compared to healthy nonsmokers, it was found that the Nrf2 protein was significantly activated in the human small airway epithelium of healthy smokers and localized in the nucleus (*p* < 0.05). The research gene expression data stored in the GEO browser (GSE 11952) was then analyzed using GEO2R to look for DEG between smokers and nonsmokers subjects. Furthermore, DEGs of smoker's vs nonsmokers were compared with genes that changed expression due to AST treatment obtained from the CTD, with the target of finding genes that were opposite in expression between the 2 datasets.

Genes with opposite expressions were then made into protein networks and clustered using STRING (string-db.org) [18]. In these genes, gene function annotations were made using the GO biological process [19, 20]. Relationships between genes and smoking-related diseases obtained from CTD. At this stage, it was expected to know the role of AST on changes in biological processes that occur in smokers and the diseases that can accompany them based on gene expression profiles.

## 3. Results

The results of DEG analysis of research data from Hübner et al. [17] stored in the GEO browser (GSE 11952), showed that there were 4912 significantly differentially expressed genes (DEGs) in the human small airway epithelium of smokers compared to nonsmokers (*p* < 0.05). If the 4192 DEGs was compared with genes or proteins whose expression changed because of AST administration obtained from CTD, then the results are as shown in Table 1.

Table 1 shows 23 Nrf2-related genes/proteins that expression regulation was opposite between smokers (against nonsmokers) with the effect of AST treatment. The effect of AST in influencing the gene expression/protein could happen directly or indirectly. In the case of it happening indirectly, the AST slows down the reaction that influences a particular gene expression/protein. For instance, slowing down LEPR mutant reaction. LEP is known to be associated with leptin receptor (LEPR) and took part in activating several intracellular signaling channels [21]. The increase of LEP in the lungs and serum is associated with potentially worsening or hastening the development of lung diseases, including acute lung injury (ALI), acute respiratory distress syndrome, chronic obstructive pulmonary disease (COPD), airway remodeling associated with asthma, and lung cancer [21]. In addition, the presence of polymorphism LEPR is known to show a statistically significant difference between lung cancer patients and controls (*p*=0.007) [22]. LEPR mutant is also known to cause kidney [23] and bone marrow fibrosis [24].

The relationship between these 23 genes and compounds found in environmental tobacco smoke (ETS) can be seen in Table 2. ETS is smoke that originated from burning tobacco products and smoke exhaled by smokers [25]. ETS consist of 40 biologically and toxicologically active compounds according to Hoffmann's list [26]. Three compounds from Hoffmann List are produced in milligrams per cigarette (tar, nicotine, and CO), while the remaining are in nanograms or micrograms level per cigarette [26, 27]. In Table 2, it could be seen that 6 out of 23 genes that undergo changes in profile expression in human small airway epithelium on smoker subjects are associated with the compounds from ETS according to the Hoffmann List. In addition to explaining how smoking can affect the expression profile of these genes, this can also clarify the potential benefits of giving AST to smokers.

**Figure 1:**
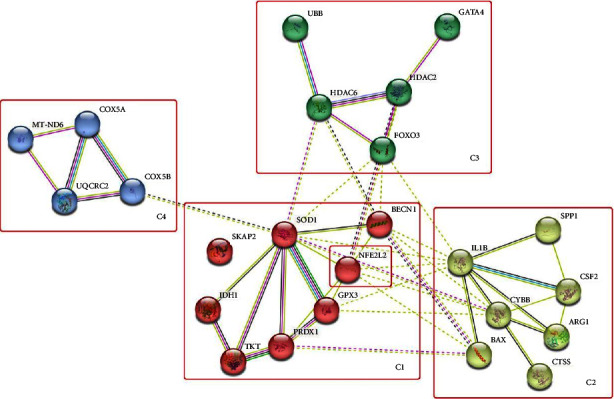
Nrf2-related protein network (NFE2L2) with reverse gene/protein expression regulation between smokers (against nonsmokers) and the effect of Astaxanthin administration.

From the 23 mentioned genes, the protein networks were made using STRING (string-db.org) as could be seen in Figure 1. There are 4 clusters with the red node as the central cluster (C1). The central cluster consists of 7 genes/proteins: SOD1, IDH1, TKT, PRDX1, GPX3 SKAP2, and BECN1 with SOD1 as central nodes. If Nrf2 (NFE2L2) was administrated into the networks, it could be seen that these genes were in the central cluster (C1). In Table 3, the annotations of the 7 (seven) genes/proteins which contain 10 (ten) groups of gene annotations (gene ontology and GO biological process) can be seen based on the smallest adjusted *p* value [28]. In Table 3, the genes/proteins can be seen to be involved in chemical and oxidative stress, reactive oxygen species (ROS), and apoptotic signaling pathway. Smoking is known to induce oxidative stress, as well as activate inflammatory response pathways, which trigger a cascade of events in which ROS production is an early but indispensable step [29]. CS is also known to induce *in vivo* epithelial cell apoptosis, however, fibrotic changes occur only after a viral exacerbation [29, 30].

Gene function annotation (GO biological process) of other clusters (C2, C3, and C4) can also be seen in Table 3. C2 cluster is related to the regulation of extrinsic apoptotic signaling pathways and the regulation of immune response. C3 cluster is related to transcription process regulation, programmed cell death, signaling receptor activity, and deacetylation reaction. While the C4 cluster is related to aerobic and cellular respiration, as well as the electron transport process in mitochondria. Based on the relationship between the central cluster and other clusters, it can be seen how chemical oxidative stress caused by ROS due to smoking activity could affect the transcription process regulation, signaling process related to apoptosis and receptor activity, as well as electron transfer process and other cellular processes.

If the 23 genes that change expression due to smoking were associated with diseases caused by smoking, it was known that all of genes were involved/associated with chronic bronchitis, adenocarcinoma of the lung, non-small-cell lung carcinoma, carcinoma, small cell lung carcinoma, type 2 diabetes mellitus, emphysema, ischemic stroke, lung diseases, and inflammation (Table 4). While the ones related to pulmonary heart disease are known to be as many as 19 genes. The interesting thing is that the 23 genes are also related to the inflammation disease category. It appears that this evidence suggests that the association between ROS and oxidative stress induced by smoking and smoking-related disease may be mediated by the inflammatory process. On the other hand, the administration of AST, thus has the potential to reduce the risk of the development of these diseases in smokers.

## 4. Discussion

Smoking activity is a major factor in various diseases, including immune-mediated inflammation disease. The concept of chronic or prolonged ROS production is central to the development of inflammatory diseases [31]. On tobacco, ROS production is mainly contributed by nicotine, the main component in tobacco. A low concentration of nicotine (0.1 *μ*M) could induce ROS to about 35%, however, a significant increase in the amount of ROS could be observed at 1 and 10 *μ*M nicotine concentrations of 54% and 80%, respectively [32]. Aside from nicotine, ROS development is also stimulated by various agents such as pollutants in ETS such as heavy metals (lead, nickel, mercury, arsenic, cadmium, chromium, and cobalt), or other organic compounds such as hydroquinone, acrylonitrile, acrolein, formaldehyde, acetaldehyde, benzene, dan benzo(a)pyrene. Reactive oxygen species (ROS) is a key signaling molecule that plays an important role in the development of inflammatory disorders. The increase in ROS generation by neutrophils polymorphonuclear (PMN) at the sites of inflammation can for example lead to endothelial dysfunction and tissue injury [31]. However, nicotine-induced neutrophil activation by nicotine is also known to be ROS-independent [33].

Some of the associations between genes that changed expression in smokers with inflammation, endothelial dysfunction, and tissue injury can be explained as follows: in Table 2, it is known that an increase in histone deacetylase2 (HDAC2) expression, which is also caused by nicotine, hydroquinone, and benzo(a)pyrene compound, happens in smokers. The increase in expression also happens on secreted phosphoprotein1 (SPP1), transketolase (TKT), cytochrome b-245 beta chain (CYBB), and peroxiredoxin 1 (PRDX1) in smoker subjects compared to nonsmokers (Table 1). In atherosclerosis, overexpression of HDAC2 in endothelial cells under proatherogenic conditions and oxidative injury suppresses the expression of Arginase2 (ARG2), which further reduces the expression of endothelial nitric oxide synthase (eNOS) [34]. Endothelial dysfunction is known to be caused by a decrease in eNOS expression. In chronic diabetic foot ulcer (DFU) an increase of HDAC2 expression also happens where dysfunctional endothelial progenitor cells (EPCs) plays a major role in inhibiting vascular complication in DFU patient [35]. Inhibition of HDAC2 is known to prevent inflammatory disorders and ROS production in EPCs with high glucose levels [35]. SPP1 is highly expressed after stimulation of oxidized low-density lipoprotein (oxLDL) and plays a role in causing inflammation of human coronary artery endothelial cells (HCAECs) [36]. High TKT expression is also associated with advanced tumor stage and TKT inhibitors promote apoptosis of lung adenocarcinoma cells and cell cycle blockade [37]. CYBB, also known as NADPH-oxidase (NOX2) is known to be involved in angiotensin II-induced hypertension and endothelial dysfunction, as well as abundantly expressed in the endothelium [38]. PRDX1 is also significantly higher in stroke patients compared to control. PRDX1 level is also higher on blood samples taken 3 and 6 hours after the stroke attack compared to the control [39].

The 23 DEGs generated from the analysis of gene expression data in the GEO browser (GSE 11952) were the genes expressed in the human small airway epithelium of smokers vs nonsmokers, where the Nrf2 protein is also significantly activated and localized in the nucleus of the same cell [17]. This can also indicate how these genes are related to Nrf2. Furthermore, if we look at the protein network in Figure 1 where Nrf2 (NFE2L2) was in the central cluster (C1), there is a strong indication that the 23 DEGs produced are related to Nrf2 (NFE2L2). In mammals, Nrf2 has long been known to function as an evolutionarily conserved intracellular defense mechanism against oxidative stress. Nrf2 has been shown to contribute to the regulation of the heme oxygenase1 (HO-1) axis, which is a strong anti-inflammatory target, and has shown a relationship with the expression of inflammatory mediators in the NF-kB pathway and macrophage metabolism through the Nrf2/antioxidant response element (ARE) system [40]. Lungs are highly vulnerable to oxidative stress-inducing factors such as infection, allergen, and pollutant such as ETS. Oxidative stress that triggers Nrd2 activation has been shown in several human respiratory diseases such as asthma and chronic obstructive pulmonary disease (COPD), or pulmonary parenchyma-related diseases such as acute respiratory distress syndrome (ARDS) and lung fibrosis [41]. In this study, it has been shown (in Table 4) the association of the 23 DEGs with these diseases and other smoking-related diseases such as pulmonary heart disease, ischemic stroke, and type 2 diabetes mellitus (T2D).

This study shows that AST could also act as a very good candidate to improve diseases related to inflammation [42]. AST is also known to increase Nrf2 and HO-1 expression in the lung, and suppress emphysema due to cigarette smoke in rats [43]. From the various previous explanations, it can be concluded that AST treatment in smokers has the potential to reduce the formation of ROS and the occurrence of oxidative stress that triggers inflammation, as well as the accompanying diseases. The potential for AST can then be confirmed through the next stage of research (e.g., clinical trials) including through observation of changes in gene expression biomarkers of the 23 DEGs.

## 5. Conclusion

From this study, we found that the 23 DEGs (smokers vs nonsmokers) in the human small airway epithelium were found to be inversely regulated by genes that changed expression due to AST treatment. Based on the GO biological process, some of these genes are known to be related to oxidative stress and ROS. AST has been confirmed to be efficacious in relieving chronic and acute inflammation in a variety of diseases, including neurodegenerative disorders, diabetes, gastrointestinal diseases, kidney inflammation, and skin and eye diseases.

## Figures and Tables

**Table 1:** DEGs in Nrf2-associated human small airway epithelium of smoker vs nonsmokers compared with changes in gene/protein expression due to AST administration taken from CTD [16].

DEG smokers vs nonsmoker (*p* < 0.05)	log_2_ (fold change)	Regulation	Astaxanthin-gene interaction	Regulation
SPP1	2.024	Up	Astaxanthin inhibits the reaction (LEPR gene mutant form results in increased expression of SPP1 mRNA)	Down
TKT	1.12	Up	Astaxanthin inhibits the reaction (LEPR gene mutant form results in increased expression of TKT mRNA)	Down
PRDX1	0.83	Up	Astaxanthin inhibits the reaction (LEPR gene mutant form results in increased expression of PRDX1 mRNA)	Down
BECN1	0.829	Up	Astaxanthin inhibits the reaction (bisphenol A results in increased expression of BECN1 protein)	Down
CYBB	0.716	Up	Astaxanthin results in decreased expression of CYBB mRNA	Down
ND6	0.715	Up	Astaxanthin inhibits the reaction (LEPR gene mutant form results in increased expression of ND6 mRNA)	Down
COX5B	0.715	Up	Astaxanthin inhibits the reaction (LEPR gene mutant form results in increased expression of COX5B mRNA)	Down
GPX3	0.699	Up	Astaxanthin inhibits the reaction (LEPR gene mutant form results in increased expression of GPX3 mRNA)	Down
UQCRC2	0.635	Up	Astaxanthin inhibits the reaction (LEPR gene mutant form results in increased expression of UQCRC2 mRNA)	Down
HDAC6	0.626	Up	Astaxanthin inhibits the reaction (lipopolysaccharide, *E coli* O55-B5 results in increased expression of HDAC6 mRNA)	Down
FOXO3	0.600	Up	Astaxanthin inhibits the reaction (LEPR gene mutant form results in increased expression of FOXO3 mRNA)	Down
GATA4	0.574	Up	Astaxanthin inhibits the reaction ((oxadiazon cotreated with butachlor) results in increased expression of GATA4 mRNA)	Down
IL1B	0.556	Up	Astaxanthin results in decreased expression of IL1B protein	Down
SKAP2	0.555	Up	Astaxanthin results in decreased expression of SKAP2 mRNA	Down
HDAC2	0.532	Up	Astaxanthin inhibits the reaction (lipopolysaccharide, *E coli* O55-B5 results in increased expression of HDAC2 protein)	Down
IDH1	0.511	Up	Astaxanthin inhibits the reaction (LEPR gene mutant form results in increased expression of IDH1 mRNA)	Down
SOD1	0.51	Up	Astaxanthin inhibits the reaction (LEPR gene mutant form results in increased expression of SOD1 mRNA)	Down
CTSS	0.471	Up	Astaxanthin results in decreased expression of CTSS mRNA	Down
BAX	0.459	Up	Astaxanthin inhibits the reaction (hydrogen peroxide results in increased expression of BAX mRNA)	Down
COX5A	0.437	Up	Astaxanthin inhibits the reaction (LEPR gene mutant form results in increased expression of COX5A mRNA)	Down
CSF2	0.428	Up	Astaxanthin inhibits the reaction (lipopolysaccharide, *E coli* O55-B5 results in increased expression of CSF2 protein)	Down
UBB	0.336	Up	Astaxanthin inhibits the reaction (LEPR gene mutant form results in increased expression of UBB mRNA)	Down
ARG1	−0.537	Down	Astaxanthin inhibits the reaction ((vehicle emissions results in increased abundance of particulate matter) which results in decreased expression of ARG1 mRNA)	Up

**Table 2:** Relationship between smoking-associated genes with biologically and toxicologically active compounds in ETS. Biologically and toxicologically active compounds data obtained from CTD [16].

Gene symbol	Chemical name	Organism	Interaction actions
ARG1	Hydroquinone	Homo sapiens	Increases^expression
BAX	Cobalt, hydroquinone, acrylonitrile, acrolein, formaldehyde, acetaldehyde, benzo(a)pyrene, chromium, nickel, arsenic, cadmium	Homo sapiens	Increases^expression
BECN1	4-cresol, hydroquinone, hydroquinone, benzo(a)pyrene	Homo sapiens	Increases^expression
CSF2	Benzene, benzo(a)pyrene	Homo sapiens	Increases^expression
CTSS	Nickel	Homo sapiens	Increases^expression
CYBB	Nickel, acetaldehyde	Homo sapiens	Increases^expression
GPX3	Selenium	Homo sapiens	Increases^expression
HDAC2	Nicotine, hydroquinone, benzo(a)pyrene	Homo sapiens	Increases^expression
IL1B	Cobalt, phenol, hydroquinone, resorcinol, formaldehyde, acetaldehyde, Benzo(a)pyrene, styrene, lead, nickel, mercury, arsenic, cadmium, selenium	Homo sapiens	Increases^expression
ND6	Formaldehyde	Homo sapiens	Increases^expression
PRDX1	Hydroquinone, benzo(a)pyrene, arsenic, cadmium	Homo sapiens	Increases^expression
SOD1	Hydroquinone, Benzo(a)pyrene, arsenic, cadmium, chromium	Homo sapiens	Increases^expression
SPP1	Acetaldehyde, benzo(a)pyrene, nickel, mercury, cadmium	Homo sapiens	Increases^expression
TKT	Hydroquinone, arsenic, selenium	Homo sapiens	Increases^expression
UBB	Cadmium	Homo sapiens	Increases^expression
ARG1	Benzo(a)pyrene	Homo sapiens	Decreases^expression

**Table 3:** Gene annotation function (GO biological process) related to Nrf2 on the central cluster C1, C2, C3, and C4.

Term	*P* value	Adjusted *P* value	Genes
*C1*			
Cellular response to chemical stress (GO:0062197)	2.119*E* − 08	3.68706*E* − 06	GPX3; PRDX1; SOD1; NFE2L2
Cellular response to oxidative stress (GO:0034599)	5.01528*E* − 08	4.36329*E* − 06	GPX3; PRDX1; SOD1; NFE2L2
Hydrogen peroxide metabolic process (GO:0042743)	9.55463*E* − 08	5.54169*E* − 06	GPX3; PRDX1; SOD1
Removal of superoxide radicals (GO:0019430)	4.7186*E* − 06	0.000200665	PRDX1; SOD1
Cellular response to superoxide (GO:0071451)	5.76623*E* − 06	0.000200665	PRDX1; SOD1
Regulation of an oxidative stress-induced intrinsic apoptotic signaling pathway (GO:1902175)	1.60221*E* − 05	0.00046464	SOD1; NFE2L2
Hydrogen peroxide catabolic process (GO:0042744)	2.19802*E* − 05	0.000546364	GPX3; PRDX1
Superoxide metabolic process (GO:0006801)	5.18203*E* − 05	0.001127092	PRDX1; SOD1
Retina homeostasis (GO:0001895)	6.57764*E* − 05	0.001271677	PRDX1; SOD1
Positive regulation of intrinsic apoptotic signaling pathway (GO:2001244)	8.13833*E* − 05	0.001416069	BECN1; SOD1

*C2*			
Negative regulation of signal transduction in absence of ligand (GO:1901099)	2.64721*E* − 05	0.004729029	CSF2; IL1B
Negative regulation of extrinsic apoptotic signaling pathway in absence of ligand (GO:2001240)	2.64721*E* − 05	0.004729029	CSF2; IL1B
Regulation of an extrinsic apoptotic signaling pathway in absence of ligand (GO:2001239)	3.95183*E* − 05	0.004729029	CSF2; IL1B
Response to organic cyclic compound (GO:0014070)	0.000184064	0.016519707	IL1B; SPP1
Regulation of T cell proliferation (GO:0042129)	0.000295584	0.016860769	ARG1; IL1B
Negative regulation of extrinsic apoptotic signaling pathway (GO:2001237)	0.000327517	0.016860769	CSF2; IL1B
Positive regulation of transport (GO:0051050)	0.000423647	0.016860769	IL1B; BAX
Neutrophil degranulation (GO:0043312)	0.000450176	0.016860769	ARG1; CYBB; CTSS
Neutrophil activation is involved in immune response (GO:0002283)	0.000461244	0.016860769	ARG1; CYBB; CTSS
Neutrophil mediated immunity (GO:0002446)	0.000469659	0.016860769	ARG1; CYBB; CTSS

*C3*			
Aggrephagy (GO:0035973)	2.02451*E* − 05	0.001871553	UBB; HDAC6
Positive regulation of transcription by RNA polymerase II (GO:0045944)	2.03436*E* − 05	0.001871553	HDAC2; UBB; GATA4; FOXO3
Negative regulation of transcription, DNA-templated (GO:0045892)	2.41389*E* − 05	0.001871553	HDAC2; UBB; FOXO3; HDAC6
Positive regulation of programmed cell death (GO:0043068)	2.8328*E* − 05	0.001871553	UBB; FOXO3; HDAC6
Positive regulation of signaling receptor activity (GO:2000273)	3.31836*E* − 05	0.001871553	HDAC2; HDAC6
Protein deacetylation (GO:0006476)	4.92876*E* − 05	0.002045828	HDAC2; HDAC6
Histone deacetylation (GO:0016575)	5.38074*E* − 05	0.002045828	HDAC2; HDAC6
Positive regulation of transcription, DNA-templated (GO:0045893)	5.80377*E* − 05	0.002045828	HDAC2; UBB; GATA4; FOXO3
Regulation of proteolysis (GO:0030162)	7.11791*E* − 05	0.002230277	HDAC2; HDAC6
Selective autophagy (GO:0061912)	8.5064*E* − 05	0.002398805	UBB; HDAC6

*C4*			
Aerobic electron transport chain (GO:0019646)	1.63813*E* − 07	5.13101*E* − 07	UQCRC2; COX5B; COX5A
Mitochondrial ATP synthesis coupled electron transport (GO:0042775)	1.71034*E* − 07	5.13101*E* − 07	UQCRC2; COX5B; COX5A
Mitochondrial electron transport, cytochrome c to oxygen (GO:0006123)	4.07582*E* − 06	8.15165*E* − 06	COX5B; COX5A
Mitochondrial electron transport, ubiquinol to cytochrome c (GO:0006122)	0.002397979	0.003596968	UQCRC2
Aerobic respiration (GO:0009060)	0.004193643	0.005032372	UQCRC2
Cellular respiration (GO:0045333)	0.008374111	0.008374111	UQCRC2

**Table 4:** Relationship between smoking-related diseases with C1, C2, C3, and C4 clusters. Data obtained from CTD (https://ctdbase.org/) [16].

Disease name	Disease ID	Disease categories	Genes
Chronic bronchitis	MESH: D029481	Pathology (process)|respiratory tract disease	SPP1; TKT; PRDX1; BECN1; CYBB; ND6; COX5B; GPX3; UQCRC2; HDAC6; FOXO3; GATA4; IL1B; SKAP2; HDAC2; IDH1; SOD1; CTSS; BAX; COX5A; CSF2; UBB; ARG1
Adenocarcinoma of lung	MESH: D000077192	Cancer	SPP1; TKT; PRDX1; BECN1; CYBB; ND6; COX5B; GPX3; UQCRC2; HDAC6; FOXO3; GATA4; IL1B; SKAP2; HDAC2; IDH1; SOD1; CTSS; BAX; COX5A; CSF2; UBB; ARG1
Non-small-cell lung carcinoma	MESH: D002289	Cancer|respiratory tract disease	SPP1; TKT; PRDX1; BECN1; CYBB; ND6; COX5B; GPX3; UQCRC2; HDAC6; FOXO3; GATA4; IL1B; SKAP2; HDAC2; IDH1; SOD1; CTSS; BAX; COX5A; CSF2; UBB; ARG1
Small cell lung carcinoma	MESH: D055752	Cancer|respiratory tract disease	SPP1; TKT; PRDX1; BECN1; CYBB; ND6; COX5B; GPX3; UQCRC2; HDAC6; FOXO3; GATA4; IL1B; SKAP2; HDAC2; IDH1; SOD1; CTSS; BAX; COX5A; CSF2; UBB; ARG1
Type 2 diabetes mellitus	MESH: D003924	Endocrine system disease|metabolic disease	SPP1; TKT; PRDX1; BECN1; CYBB; ND6; COX5B; GPX3; UQCRC2; HDAC6; FOXO3; GATA4; IL1B; SKAP2; HDAC2; IDH1; SOD1; CTSS; BAX; COX5A; CSF2; UBB; ARG1
Emphysema	MESH: D004646	Pathology (process)	SPP1; TKT; PRDX1; BECN1; CYBB; ND6; COX5B; GPX3; UQCRC2; HDAC6; FOXO3; GATA4; IL1B; SKAP2; HDAC2; IDH1; SOD1; CTSS; BAX; COX5A; CSF2; UBB; ARG1
Ischemic stroke	MESH: D000083242	Cardiovascular disease|nervous system disease	SPP1; TKT; PRDX1; BECN1; CYBB; ND6; COX5B; GPX3; UQCRC2; HDAC6; FOXO3; GATA4; IL1B; SKAP2; HDAC2; IDH1; SOD1; CTSS; BAX; COX5A; CSF2; UBB; ARG1
Lung diseases	MESH: D008171	Respiratory tract disease	SPP1; TKT; PRDX1; BECN1; CYBB; ND6; COX5B; GPX3; UQCRC2; HDAC6; FOXO3; GATA4; IL1B; SKAP2; HDAC2; IDH1; SOD1; CTSS; BAX; COX5A; CSF2; UBB; ARG1
Pulmonary heart disease	MESH: D011660	Cardiovascular disease	SPP1; BECN1; CYBB; ND6; COX5B; GPX3; UQCRC2; HDAC6; FOXO3; GATA4; IL1B; IDH1; SOD1; CTSS; BAX; COX5A; CSF2; UBB; ARG1
Inflammation	MESH: D007249	Pathology (process)	SPP1; TKT; PRDX1; BECN1; CYBB; ND6; COX5B; GPX3; UQCRC2; HDAC6; FOXO3; GATA4; IL1B; SKAP2; HDAC2; IDH1; SOD1; CTSS; BAX; COX5A; CSF2; UBB; ARG1

## Data Availability

The data used to support the findings of the study are available on the database mentioned in the manuscript, as it used secondary data from the Gene Omnibus Ontology (GEO) database and Comparative Toxicogenomics Database (CTD; https://ctdbase.org/).
